# Isolating the direct effects of growth hormone on lifespan and metabolism in mice

**DOI:** 10.1111/acel.14412

**Published:** 2024-11-20

**Authors:** Alexander Tate Lasher, Kaimao Liu, Michael P Fitch, Liou Y. Sun

**Affiliations:** ^1^ Department of Biology University of Alabama at Birmingham Birmingham Alabama USA

**Keywords:** aging, growth hormone, lifespan, longevity regulation, metabolic health, mouse models

## Abstract

Prior studies have shown that interrupting the growth hormone/insulin‐like growth factor‐I (GH/IGF‐I) signaling axis extends laboratory mouse lifespan, but confounding effects of additional gene or hormone deficiencies that exist in commonly used models of GH/IGF‐I interruption obscure the specific effect of GH on longevity. We address this issue by using mice with a specific knockout of the GH gene and show that both males and females on a mixed genetic background display extended lifespans resulting from GH deficiency. Our physiological assessment of these mice revealed that in addition to weighing significantly less and displaying significantly greater body fat (as a percentage of body weight), GH deficient mice display significant impairments in glucose metabolism and preferential fat utilization. These data provide strong evidence that GH deficiency is directly responsible for the altered nutrient utilization and extended lifespan that is commonly observed in mouse models of GH/IGF‐I interruption.

## Graphical abstract

Previous studies suggest that disrupting the growth hormone (GH) pathway can extend mouse lifespan, but confounding factors often cloud this relationship. To clarify, we used mice with a specific GH gene knockout and found that both males and females had extended lifespans solely due to GH deficiency. Physiological assessments showed these mice were lighter, had higher body fat percentages, and exhibited impaired glucose metabolism. Our results strongly indicate that GH deficiency directly impacts nutrient utilization and contributes to increased lifespan.

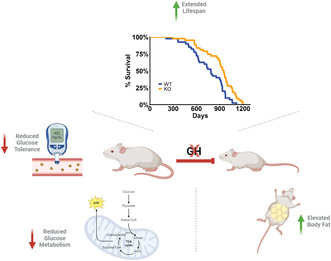

**Abbreviations**
CR: calorie restriction
FOx: fat oxidation
GH: growth hormone
GHR: growth hormone receptor
GHRH: growth hormone‐releasing hormone
GOx: glucose oxidation
GTT: glucose tolerance test
IGF‐1 (or IGF‐I): insulin‐like growth factor 1 (or insulin‐like growth factor I)
RQ: respiratory quotient

## 1 INTRODUCTION

The somatotrophic axis, involving GH‐releasing hormone (GHRH) and GH from the hypothalamus and pituitary, significantly influences the lifespan of laboratory mice. Studies show that mice with genetic disruptions in this axis display extreme longevity (Bartke, 2021; Bartke et al., 2013), highlighting GH's role as a lifespan regulator. However, “GH‐deficient” mice often have other genetic and hormonal defects, confounding GH's direct impact. Ames dwarf and Snell dwarf mice, both GH‐deficient and long‐lived (Brown‐Borg et al., 1996; Flurkey et al., 2001), also lack prolactin and thyroid‐stimulating hormone, muddling GH's specific effects. Additionally, long‐lived GHRH knockout (Sun et al., 2013) and GHRH receptor deficient (Flurkey et al., 2001) mice are not true models of isolated GH deficiency due to the absence of GHRH and its potent extrapituitary effects (Granata, 2016). Further, long‐lived GH receptor knockout (GHRKO) mice display dramatically elevated plasma GH (Coschigano et al., 2000; Zhou et al., 1997) potentially leading to nonspecific GH action.

These caveats are biologically relevant. Calorie restriction (CR), which extends lifespan (Swindell, 2012), boosts longevity in mixed genetic background Ames dwarf (Bartke et al., 2001) and GHRH‐KO mice (Sun et al., 2013) but does not affect GHRKO lifespan (Bonkowski et al., 2006) or the lifespan of inbred Ames dwarf mice (Garcia et al., 2008). Further, recent findings show that double mutant GHRH‐KO × GHRKO mice have significantly different circulating IGF‐1, leptin, and adiponectin levels compared to WT, GHRKO, or GHRH‐KO mice (Icyuz et al., 2021). These suggest GH resistance and GH deficiency influence lifespan or healthspan through different mechanisms. These different phenotypes bring the specific role of GH into question. To bridge this knowledge gap, we assessed lifespan and metabolism in mice with a targeted knockout of the GH gene.

## 2 RESULTS

**FIGURE 1:**
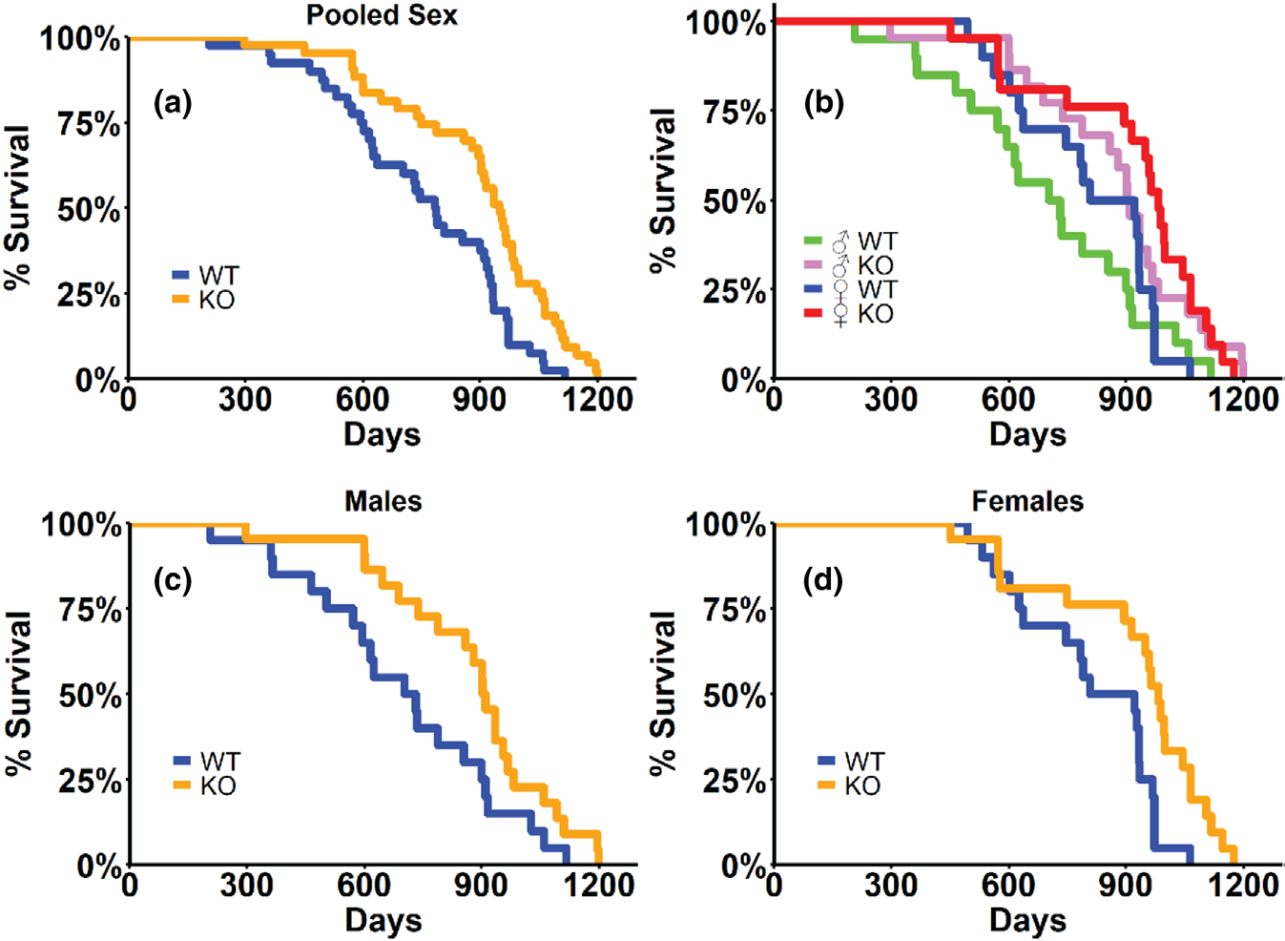
Isolated GH deficiency extends lifespan. Kaplan–Meier survival curves for pooled sexes (a), all groups (b), male mice (c) and female mice (d). Statistical analyses of lifespans are provided in Table 1. WT = wild type; KO = GH knockout mice. *N* = 20 male WT; *n* = 22 male KO; *n* = 20 female WT; *n* = 21 female KO mice.

GH knockout (KO) mice displayed no *Gh* expression in the pituitary and dramatic reductions in liver *Igf1* expression (Figure S1a–d). KO mice on a mixed C57BL/6N × C57BL/6J × BALB/cByJ genetic background maintained under specific pathogen‐free conditions with ad‐lib access to standard rodent diet (NIH‐31) and water displayed a 21% extension in median lifespan over WT littermates (786 days vs. 949 days; Figure 1a), a significant extension in life (*p* = 0.0011, logrank test; Table 1). Cox proportional hazard analysis revealed significantly lower hazard ratios (*p* = 0.0012; Table 1), and survival at the 75th (*p* = 0.0023) and 90th (*p* = 0.0303) percentiles were significantly higher in KO mice when assessed by quantile regression (Wang et al., 2004) (Table 1). When sexes were analyzed separately, KO males displayed a 27% extension in median lifespan over WT males (716 days vs. 906 days; Figure 1b,c), a significant extension (*p* = 0.0380, logrank test; *p* = 0.0427, Cox proportional hazard; Table 1). KO females displayed a more modest, yet still statistically significant, 14% extension in lifespan (864 days vs. 982 days; Figure 1b,d) over WT females (*p* = 0.0038, logrank test; *p* = 0.0041 Cox proportional hazard; Table 1). We did not detect any differences in the lifespans of males and females within the same genotype (Table 1).

**TABLE 1:** Statistical analysis of lifespans.

Sex	Group	Median Lifespan	Logrank *p* value	Cox Proportional Hazard		Maximal Lifespan	
				Hazard Ratio	*p* Value	75th *p* value	90th *p* value
Pooled	WT (*n* = 40)	786 days	0.0010	1	0.0012	0.0023	0.0303
	KO (*n* = 43)	949 days		0.4695			
Male	WT (*n* = 20)	716 days	0.0374	1	0.0415	NA	NA
	KO (*n* = 22)	906 days		0.5209			
Female	WT (*n* = 20)	864 days	0.0037	1	0.0041	NA	NA
	KO (*n* = 21)	982 days		0.3520			
Male WT (*n* = 20)		716 days	0.3588	1	0.417	NA	NA
Female WT (*n* = 20)		864 days		0.7671			
Male KO (*n* = 22)		906 days	0.6230	1	0.623	NA	NA
Female KO (*n* = 21)		982 days		0.8562			

*Note*: Maximal lifespan was analyzed for pooled sexes as the low sample size that results from stratifying sexes prevented meaningful statistical comparisons.

**FIGURE 2:**
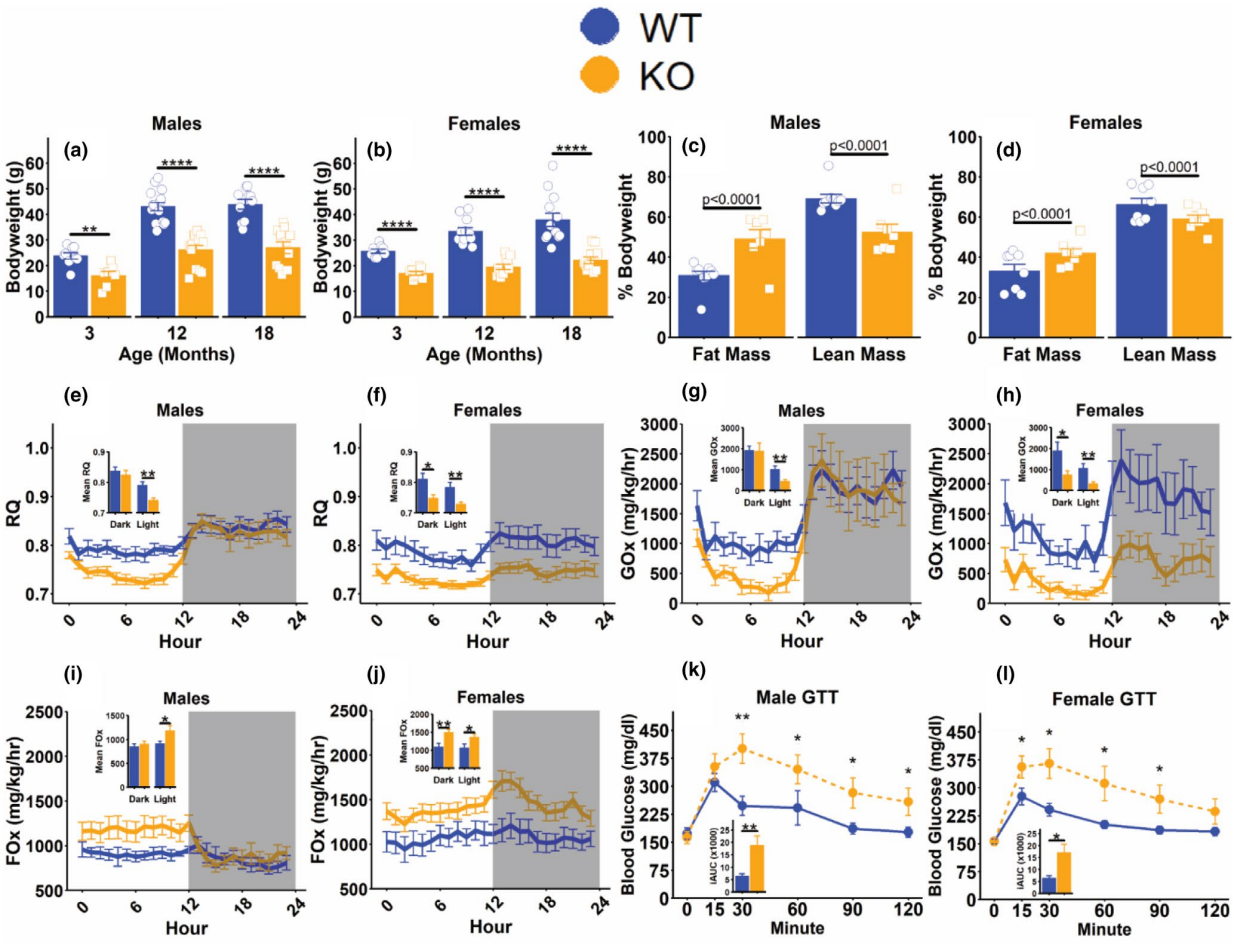
Physiological features of 18‐month‐old mice with isolated GH deficiency. Bodyweight of male (a) and female (b) mice at the indicated ages. Fat and lean mass as percentages of total bodyweight in male (c) and female (d) mice. 24‐h respiratory quotient (RQ; VCO_2_/VO_2_) measured during indirect calorimetry in male (e) and female (f) mice, with insets representing mean RQ during the dark or light phases as indicated. 24‐h glucose oxidation (GOx) normalized to bodyweight in male (g) and female (h) mice with insets representing mean GOx during dark or light phases as indicated. 24‐h fat oxidation (FOx) normalized to bodyweight in male (i) and female (j) mice with insets representing mean FOx during dark or light phases as indicated. 1 g/kg glucose tolerance test in overnight fasted male (k) and female (l) mice, with insets representing incremental area under the curve (iAUC) analysis where only values above baseline are considered. Data presented as mean ± SEM with points representing individual mice. *p < 0.05; **p < 0.01; ****p < 0.0001 as determined by two‐tailed *t*‐test with the welch correction applied or by Mann–Whitney U‐test (g, h, k, l). P‐values presented represent the main effect of genotype on fat or lean mass determined by ANCOVA with bodyweight as a covariate (c, d). *N* = 7–13 (a–d), 9–15 (e–j), or 9–13 (k, l) per group.

Physiological analysis of a separate cohort of mice showed reduced body weights and disproportionately higher body fat (Figure 2a–d), consistent with previous reports (List et al., 2019), as well as lower food consumption and cage activity in KO mice (Figure S1e–h). Nutrient utilization and metabolic rate assessed by indirect calorimetry revealed significantly lower respiratory quotient (RQ) in male KOs during the light cycle and during both light/dark cycles in female KOs (Figure 2e,f) as well as significantly lower energy expenditure (Figure S1i–l). RQ varies inversely with fat oxidation (Zurlo et al., 1990), prompting us to explore fuel utilization more fully in these mice. Calculation of glucose or fat oxidation rates (GOx or FOx) as previously (Lasher & Sun, 2023) revealed marked reduction in light cycle male KO GOx (Figure 2g) and significant elevations in light cycle FOx (Figure 2i). The reduced GOx (Figure 2h) and elevated FOx (Figure 2j) were observed during light/dark cycles for female KOs. These measures indicate KO mice rely on fat oxidation and less on glucose oxidation to meet energy demands. Further supporting the disrupted glucose metabolism in KO mice is the impaired glucose tolerance, evidenced by elevated glycemia and elevated area under the curve in males and females following a glucose challenge (Figure 2k,l). While the impaired glucose tolerance observed here is consistent with a previous report (List et al., 2019), insulin sensitivity was enhanced only in male KOs and unchanged in female mice (Figure S1m,n).

## 3 DISCUSSION

In this study, we show that targeted GH‐KO mice live longer. While the differences in lifespan we observed between KO and WT mice were significant, they are lesser in magnitude than the 40+% extensions reported in other models of somatotrophic disruption (Bartke et al., 2001; Bonkowski et al., 2006; Brown‐Borg et al., 1996; Flurkey et al., 2001; List et al., 2011; Sun et al., 2013). While our median KO mouse lifespan of 949 days is consistent with the 931 (Sun et al., 2013) day and the 1004 (Sun et al., 2017) day median lifespans observed by us in other models of somatotrophic interruption, our median female WT survival of 864 days is considerably longer than in these previous reports and may have reduced the magnitude of difference in median survival between KO and WT mice. The 38% difference in median lifespan between our shortest‐ and longest‐lived groups in this study, while certainly an extension, remains below the previously described 40% enhancement. This suggests that while GH deficiency clearly contributes to lifespan extension, an additive effect of additional gene/hormone deficiencies on lifespan may also exist. The mixed genetic background we employed may also be a contributor to these observations, particularly in our WT females. It has been shown that a more than three‐fold difference in lifespan can exist between different inbred mouse strains (Selman & Swindell, 2018; Yuan et al., 2009). These challenges are not overcome when genetic background is mixed either. Past work in CR has shown similar variation in lifespans when different recombinant inbred mice are used (Liao et al., 2010) and it is possible that a similar phenomenon may be in effect for GH deficiency. Indeed, when Ames dwarf mice are maintained on a C57BL/6J background, 40% CR shortens lifespan (Garcia et al., 2008) while 30% CR extends the lifespan of Ames dwarf mice on a heterogenous background (Bartke et al., 2001; Mattison et al., 2000).

GH expression can be detected and induced in nonpituitary tissue in response to DNA damage (Chesnokova et al., 2013), and this nonpituitary GH has recently been shown to act in paracrine manner to trigger senescent cell reentry into the cell cycle and result in further DNA damage accumulation (Chesnokova et al., 2024). As the accumulation of DNA damage is characteristic of aged cells/tissue (López‐Otín et al., 2013), it is possible that GH deletion confers a protection from the accumulation of DNA damage, resulting in the longevity and unique physiology of our GH knockout mice. Further study investigating how the local effects of GH ultimately affect mammalian lifespan represent a promising avenue for understanding how the GH/IGF‐I axis controls the aging process.

Our findings show a reduced reliance on glucose metabolism in GH disrupted mice, consistent with other reports (Brooks et al., 2007; Icyuz et al., 2020; Westbrook et al., 2009). As defects in carbohydrate metabolism have been implicated in several age‐associated diseases (Brewer et al., 2016), a lower dependency on glucose metabolism may provide resistance to these conditions in somatotrophic‐interrupted mice. In line with this reasoning, lifespan‐extending dietary interventions such as CR, ketogenic, and isocaloric high‐fat diets also reduce glucose utilization in rodents (Duffy et al., 1989; Roberts et al., 2017; Shi et al., 2021). Glucose intolerance (assessed by GTT) has been reported in GHRKO (Duran‐Ortiz et al., 2021) and GH‐KO mice (List et al., 2019) (Figure 2k,l) but not in dwarf (Hill et al., 2016) or GHRH‐KO mice (Icyuz et al., 2020; Sun et al., 2013), highlighting that while glucose utilization is consistently reduced in these models, glucose uptake is not. GH is a known antagonist of insulin action, impairing the ability of insulin to stimulate glucose uptake and contributing to insulin resistance (Sharma et al., 2020). Previous studies in GH overexpressing mice support this, as reduced insulin sensitivity has been reported in these mice (Dominici et al., 1998). While enhanced insulin sensitivity generally accompanies the metabolic changes observed in somatotrophic‐interrupted mice (Liu et al., 2004; Wiesenborn et al., 2014; Zhang et al., 2020), here we only detected this in male GH‐KO mice. This conflicts with a previous study in this model where insulin sensitivity was increased in both males and females (List et al., 2019). The different genetic backgrounds and ages of mice used between this previous report and our study may contribute to this discrepancy, genetic background plays a major role in insulin sensitivity (Parks et al., 2015).

In summary, our study reveals that isolated GH deficiency increases both average and maximum lifespan, characterized by excess adiposity and impaired glucose metabolism, highlighting GH as a significant regulator of mammalian longevity.

## 4 METHODS

The methods section can be found in the Appendix S1.

## AUTHOR CONTRIBUTIONS

ATL took the lead in writing the manuscript. ATL and LYS designed experiments and analyzed data. KL collected data. KL and MF maintained the mouse colony. LYS conceived the study, secured funding, and supervised overall direction. All authors contributed critical feedback that shaped the study and manuscript.

## FUNDING INFORMATION

The authors received research support from the National Institutes of Health AG082327and AG057734 to LYS. The UAB Small Animal Phenotyping Core supported by the NIH Nutrition & Obesity Research Center P30DK056336, Diabetes Research Center P30DK079626, and the UAB Nathan Shock Center P30AG050886A.

## CONFLICT OF INTEREST STATEMENT

All contributing authors declare no conflict of interest.

## Supporting information


Appendix S1.

## Data Availability

All data used to generate the statistical analyses and figures in this manuscript are available from the corresponding author upon reasonable request.
