# Supplementary material for: Isolating the direct effects of growth hormone on lifespan and metabolism in mice
Source: Aging Cell. 2024 Nov 20;23(12):e14412. doi: 10.1111/acel.14412 (PMC11634705; doi:10.1111/acel.14412)
Supplement: Supplementary file 1 — Appendix S1. [file ACEL-23-e14412-s001.docx]

**Methods**

*Mice*

Mice with a deletion of the GH genomic sequence generated by Regeneron Pharmaceuticals using the VelociGene (Valenzuela et al., 2003) strategy were obtained as live mice from the KOMP Repository ([www.KOMP.org](http://www.KOMP.org)) at the University of California, Davis (Stock #: 047836-UCD). These mice have previously been characterized as lacking circulating GH (List et al., 2019) and are referred to as “KO” mice. Briefly, the VelociGene method (Valenzuela et al., 2003) was used to remove the entire coding region of the mouse GH gene with a LacZ/neomycin resistance expression-selection cassette. Heterozygous mice carrying this mutation were bred to a ubiquitous Cre deleter mouse line for recombination of the LoxP-flanked neomycin cassette before being received by our lab. Genetic deletion of the *Gh* gene, coding for GH, was validated by RT-qPCR as detailed below. Mice were housed at up to 7 individuals per cage in a specific pathogen‐free facility maintained on a standard 12‐h light and 12‐h dark cycle at 20–23°C. Original mice were obtained on the C57BL/6N genetic background. C57BL/6N males carrying the mutation were mated with C57BL/6J WT (JAX stock #000664) and with BALB/cByJ WT (JAX stock #001026) females. The offspring of the C57BL/6N x C57BL/6J mating were bred with the offspring of the C57BL/6N x BALB/cByJ mating to produce mice on a mixed C57BL/6N x C57BL/6J x BALB/cByJ genetic background. This was done, as our group previously has (Icyuz et al., 2020; Lasher & Sun, 2023; Nagarajan et al., 2024; Zhang et al., 2020), to increase genetic diversity, increase fecundity, and reduce artifactual findings that arise from utilizing mice on a homogenous genetic background. These mixed-background mice were used for all experiments. All mice had *ad‐libitum* access to standard rodent chow (NIH‐31 Rat and Mouse diet, 18% protein, 4% fat) and drinking water. All experimental protocols utilizing live animals were approved by the University of Alabama at Birmingham institutional animal care and use committee.

*Metabolic Studies*

All mice were 18 months old at the time of metabolic assessment. Body composition was assessed by quantitative magnetic resonance using an EchoMRI body composition analyzer (EchoMRI, Houston, TX). Data collected included body fat mass, lean mass, and total body weight. The fat mass and lean mass were visualized as percentages of total body weight. Grams of fat mass or lean mass were statistically compared with body weight as a covariate to account for the dramatic differences in body size of our WT and KO mice while appropriately controlling for the allometric relationship between fat or lean mass and total body weight. Body composition assessment was carried out by the University of Alabama at Birmingham Small Animal Phenotyping Core Facility.

Nutrient utilization and metabolic rate were assessed by indirect calorimetry. Mice were individually housed in comprehensive lab animal monitoring system (CLAMS; Columbus Instruments, Columbus, OH) chambers. This system continuously collects oxygen consumption (VO_2_) and carbon dioxide production (VCO_2_) data every 9 min for each individually housed mouse. This system also monitors mouse activity and food consumption by detecting beam breaks in an infrared laser grid within each chamber and food weight changes in the feeder-balance systems of each chamber. For food consumption, only periods where the feeder-balance system did not register gains were considered (such as when the mouse sits on the feeder). This was done to avoid quantitating the mouse’s bodyweight as food consumed, however it also ignores food that is consumed if the mouse happens to feed while seated in the feeder. For assessment of mouse activity, only laser beam breaks that were different from the previously broken beam were considered, as this represents movement rather than repetitive stationary activity (i.e. grooming). Mice were individually housed in calorimetry chambers for a period of 48 hours, the first 24 of which were considered acclimation and were not included in data analysis. Respiratory quotient, glucose oxidation, and fat oxidation were calculated as VCO_2_/VO_2_, 4.57(VCO_2_)–3.23(VO_2_), and 1.69(VO_2_)–1.69(VCO_2_), respectively as previously described (Lasher & Sun, 2023; Nagarajan et al., 2024; Simonson & DeFronzo, 1990), and were normalized to body weight. Energy expenditure was calculated as VO_2_(3.815 + 1.232(VCO_2_/VO_2_)) according to the work of Graham Lusk (1928). ANCOVA, with bodyweight as a covariate, was used to compare mean energy expenditure between WT and KO groups to properly account for the effect bodyweight has on this metric (Tschöp et al., 2011).

Glucose tolerance tests (GTTs) and insulin tolerance tests (ITTs) were carried out in 16-hour fasted mice (for GTT) or in unfasted mice with food removed immediately before testing (for ITT). Blood glucose was taken immediately prior (“minute 0”) and at the indicated time points following an intraperitoneal injection of 1g/kg glucose (for GTT) or 0.7U/kg Humulin-R (Eli-Lilly, Indianapolis, IN, for ITT) prepared in 0.9% saline. A PRESTO handheld glucometer (AgaMatrix, Salem, NH) was used to measure blood glucose.

*RNA Extraction and Reverse Transcription Quantitative Polymerase Chain Reaction (RT-qPCR)*

RT-qPCR was used to validate our model instead of assessing circulating GH levels. 4-month-old mice were sacrificed by carbon dioxide induced anesthesia followed by cervical dislocation. The pituitary and liver from these mice were dissected and RNA was extracted from these tissues using a RNeasy Plus Mini Kit (Qiagen, Hilden, Germany) following manufacturer instructions. Equivalent amounts of total RNA were reverse transcribed using random hexamers and M-MuLV Reverse Transcriptase (New England Biolabs, Ipswich, MA) according to the recommended manufacturer protocol. RT-qPCR reactions were carried out using a QuantStudio 3 instrument and Luna Universal qPCR Master Mix (New England Biolabs, Ipswich, MA) using the following primers: Gh F 5’-GCTCTGATGCAGGAGCTGGA-3’; Gh R 5’-CGCAGGTAGGTCTCCGCTTT-3’; Igf1 F 5’-CATAGTACCCACTCTGACCTGCTGTG-3’; Igf1 R 5’-CGCCAGGTAGAAGAGGTGTGAAGAC-3’; Actb F: 5’-CTCCCTGGAGAAGAGCTATGA-3’; Actb R 5’-CAGGATTCCATACCCAAGAAGG-3’. Fold change was calculated using the 2^-ddCt^ method with *Actb* used as a normalizer. PCR products were separated on acrylamide gel where no signal was detected during RT-qPCR to verify the absence of a target.

*Statistical analysis*

The Log-rank test and Cox proportional hazard testing were employed to assess overall survival between groups. To compare maximal lifespan, a quantile regression assessment described by Wang and colleagues (Wang et al., 2004) where the proportion of mice alive at the 75^th^ and 90^th^ percentiles of survival were compared. Group means were compared using the unpaired two-tailed t-test with the welch correction applied, the Mann-Whitney U-test (where data were non-parametric), or by factorial repeated measure ANOVA as indicated in the figure legends. To assess energy expenditure and body composition, ANCOVA with body weight treated as a covariate was used to control for the effect of body mass on metabolic rate. For all statistical tests significance was established at *p* < 0.05. Analyses were carried out and figures were generated using the R programming language.

**Supplemental Figure Legends**

**Supplemental figure 1**.

General strategy for the generation of GH deficient mice (a). Relative mRNA expression (normalized to *Actb*) of *Gh* in the pituitary and *Igf1* in the liver of male (b) and female (c) mice. Acrylamide gel electrophoresis of PCR product confirms the absence of *Gh* in KO mice (representative gel, d). Cumulative food intake (e, f) and home cage activity (g, h) recorded during indirect calorimetry in male and female mice as indicated. Energy expenditure (EE) calculated during indirect calorimetry in males (i) and ANCOVA analysis for mean EE measurements, with bodyweight as a covariate, recorded during indirect calorimetry experiments in males (j). EE calculated during indirect calorimetry in females (k) and ANCOVA analysis for mean EE measurements, with bodyweight as a covariate, recorded during indirect calorimetry experiments in females (l). 0.7 U/kg insulin tolerance test in males (m) and females (n), with insets representing net area under the curve (nAUC) analysis with no baseline corrections made. Data presented as mean ± SEM with points representing individual mice. *p<0.05; **p<0.01; ***p<0.001. P-values determined by student’s t-test (b, d), two-way repeated measure ANOVA (e-h), ANCOVA (j, l), or Mann-Whitney U-test (m, n). N=3 per group (b, c) or n=9-15 per group (e-n).

**Supplemental figure 1**


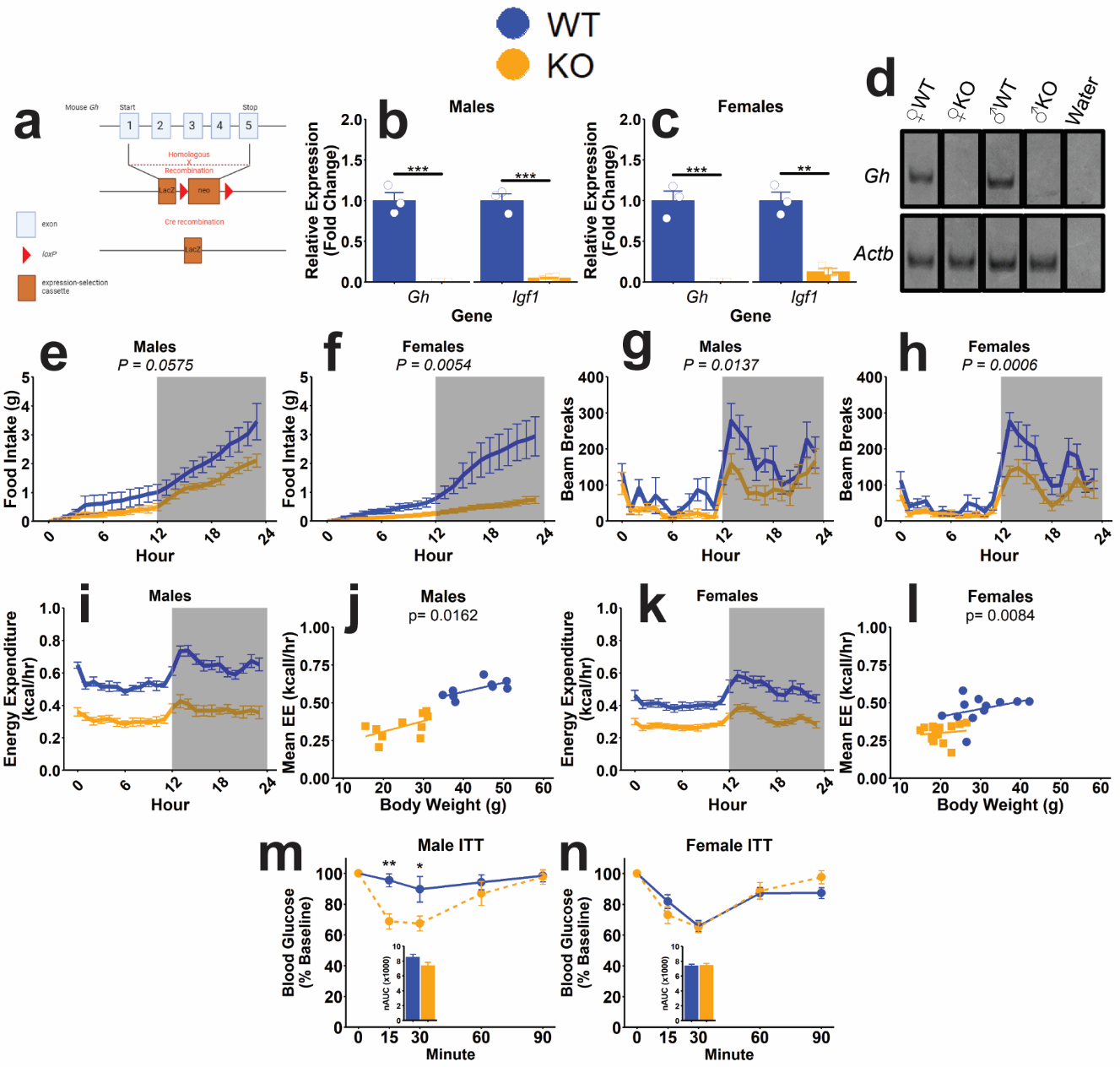


**Supplemental figure 2**

Uncropped acrylamide gel presented in supplemental figure S1d. 167bp *Gh* PCR product or 114bp *Actb* PCR product. Final two lanes are “no template controls” where nuclease-free water was used as template for PCR reactions with the primer pairs for *Gh* or *Actb* as indicated.


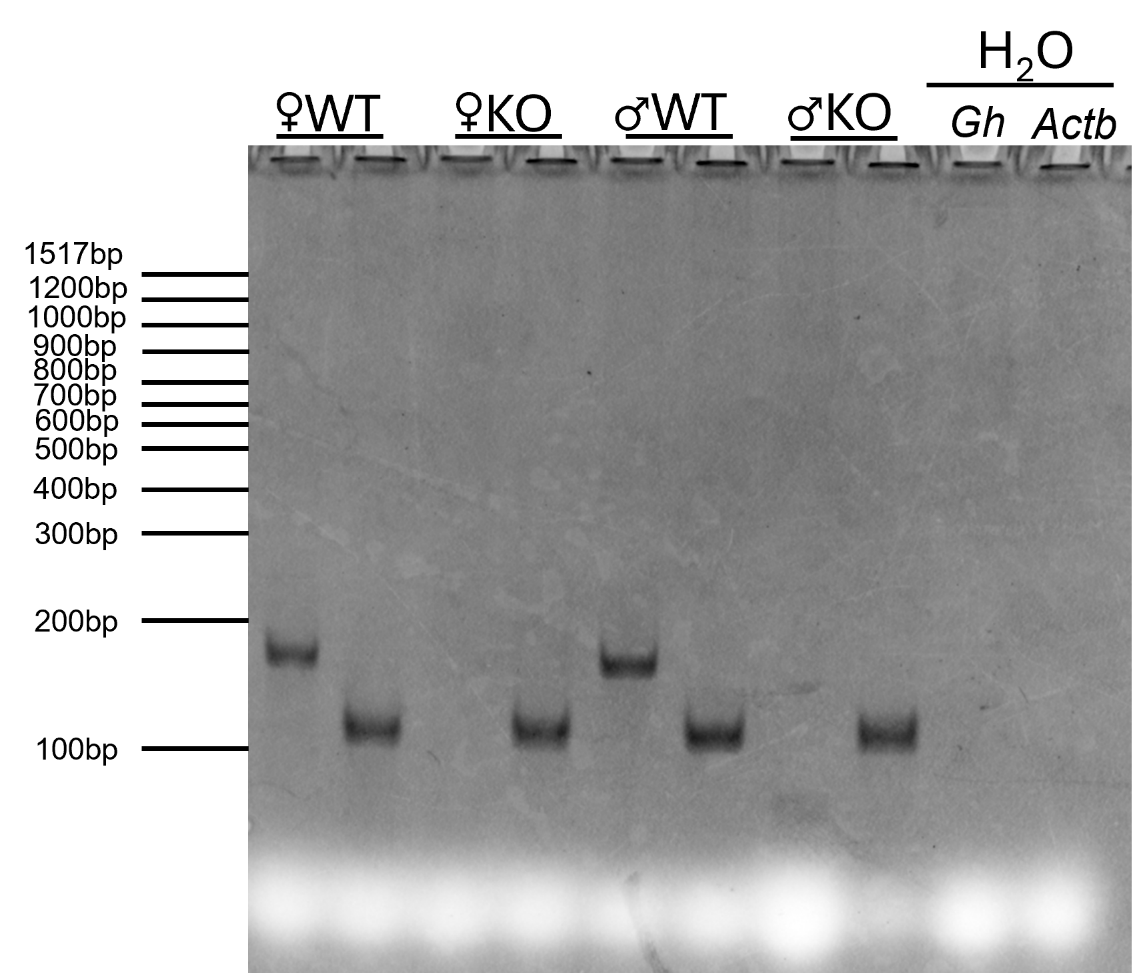


**References**

Icyuz, M., Fitch, M., Zhang, F., Challa, A., & Sun, L. Y. (2020). Physiological and metabolic features of mice with CRISPR/Cas9-mediated loss-of-function in growth hormone-releasing hormone. *Aging (Albany NY)*, *12*(10), 9761-9780. <https://doi.org/10.18632/aging.103242>

Lasher, A. T., & Sun, L. Y. (2023). Distinct physiological characteristics and altered glucagon signaling in GHRH knockout mice: Implications for longevity. *Aging Cell*, e13985. <https://doi.org/10.1111/acel.13985>

List, E. O., Berryman, D. E., Buchman, M., Jensen, E. A., Funk, K., Duran-Ortiz, S., Qian, Y., Young, J. A., Slyby, J., McKenna, S., & Kopchick, J. J. (2019). GH Knockout Mice Have Increased Subcutaneous Adipose Tissue With Decreased Fibrosis and Enhanced Insulin Sensitivity. *Endocrinology*, *160*(7), 1743-1756.

Lusk, G. (1928). *The Elements of the Science of Nutrition*. W. B. Saunders. <https://books.google.com/books?id=NNXBuQEACAAJ>

Nagarajan, A., Lasher, A. T., Morrow, C. D., & Sun, L. Y. (2024). Long term methionine restriction: Influence on gut microbiome and metabolic characteristics. *Aging Cell*, *23*(3), e14051. <https://doi.org/10.1111/acel.14051>

Simonson, D. C., & DeFronzo, R. A. (1990). Indirect calorimetry: methodological and interpretative problems. *Am J Physiol*, *258*(3 Pt 1), E399-412. <https://doi.org/10.1152/ajpendo.1990.258.3.E399>

Tschöp, M. H., Speakman, J. R., Arch, J. R., Auwerx, J., Brüning, J. C., Chan, L., Eckel, R. H., Farese, R. V., Jr., Galgani, J. E., Hambly, C., Herman, M. A., Horvath, T. L., Kahn, B. B., Kozma, S. C., Maratos-Flier, E., Müller, T. D., Münzberg, H., Pfluger, P. T., Plum, L., . . . Ravussin, E. (2011). A guide to analysis of mouse energy metabolism. *Nat Methods*, *9*(1), 57-63. <https://doi.org/10.1038/nmeth.1806>

Valenzuela, D. M., Murphy, A. J., Frendewey, D., Gale, N. W., Economides, A. N., Auerbach, W., Poueymirou, W. T., Adams, N. C., Rojas, J., Yasenchak, J., Chernomorsky, R., Boucher, M., Elsasser, A. L., Esau, L., Zheng, J., Griffiths, J. A., Wang, X., Su, H., Xue, Y., . . . Yancopoulos, G. D. (2003). High-throughput engineering of the mouse genome coupled with high-resolution expression analysis. *Nat Biotechnol*, *21*(6), 652-659. <https://doi.org/10.1038/nbt822>

Wang, C., Li, Q., Redden, D. T., Weindruch, R., & Allison, D. B. (2004). Statistical methods for testing effects on "maximum lifespan". *Mech Ageing Dev*, *125*(9), 629-632. <https://doi.org/10.1016/j.mad.2004.07.003>

Zhang, F., Icyuz, M., Liu, Z., Fitch, M., & Sun, L. Y. (2020). Insulin sensitivity in long-lived growth hormone-releasing hormone knockout mice. *Aging (Albany NY)*, *12*(18), 18033-18051. <https://doi.org/10.18632/aging.103588>
